# Delving into the Complexity of Analogical Reasoning: A Detailed Exploration with the Generalized Multicomponent Latent Trait Model for Diagnosis

**DOI:** 10.3390/jintelligence12070067

**Published:** 2024-07-18

**Authors:** Eduar S. Ramírez, Marcos Jiménez, Víthor Rosa Franco, Jesús M. Alvarado

**Affiliations:** 1 Department of Psychobiology and Behavioral Sciences Methods, Faculty of Psychology, Complutense University of Madrid, Campus Somosaguas, Carretera De Húmera, s/n, 28006 Madrid, Spain; 2 Faculty of Health Sciences, Universidad Villanueva, Calle Costa Brava 2, 28034 Madrid, Spain; 3 Department of Social Psychology and Methodology, Faculty of Psychology, Autonomous University of Madrid, Cantoblanco University City, 28049 Madrid, Spain; marcosjnezhquez@gmail.com; 4 Department of Psychology, Sao Francisco University, Campinas 13045-510, Brazil; vithorfranco@gmail.com

**Keywords:** analogical reasoning, response processes, multicomponent analysis, LLTM, MLTM-D, GMLTM-D

## Abstract

Research on analogical reasoning has facilitated the understanding of response processes such as pattern identification and creative problem solving, emerging as an intelligence predictor. While analogical tests traditionally combine various composition rules for item generation, current statistical models like the Logistic Latent Trait Model (LLTM) and Embretson’s Multicomponent Latent Trait Model for Diagnosis (MLTM-D) face limitations in handling the inherent complexity of these processes, resulting in suboptimal model fit and interpretation. The primary aim of this research was to extend Embretson’s MLTM-D to encompass complex multidimensional models that allow the estimation of item parameters. Concretely, we developed a three-parameter (3PL) version of the MLTM-D that provides more informative interpretations of participant response processes. We developed the Generalized Multicomponent Latent Trait Model for Diagnosis (GMLTM-D), which is a statistical model that extends Embretson’s multicomponent model to explore complex analogical theories. The GMLTM-D was compared with LLTM and MLTM-D using data from a previous study of a figural analogical reasoning test composed of 27 items based on five composition rules: figure rotation, trapezoidal rotation, reflection, segment subtraction, and point movement. Additionally, we provide an R package (GMLTM) for conducting Bayesian estimation of the models mentioned. The GMLTM-D more accurately replicated the observed data compared to the Bayesian versions of LLTM and MLTM-D, demonstrating a better model fit and superior predictive accuracy. Therefore, the GMLTM-D is a reliable model for analyzing analogical reasoning data and calibrating intelligence tests. The GMLTM-D embraces the complexity of real data and enhances the understanding of examinees’ response processes.

## 1. Introduction

The component models of Item Response Theory (IRT), starting with the Fischer’s Linear Logistic Test Model (LLTM) (Fischer 1973), represented a significant advancement in the measurement of intelligence. This breakthrough brought about a paradigm shift to understand the specific aspects that an individual masters or fails to master by directly estimating the difficulty components embedded in the items of a test. This focus on the response processes of the examinees was then expanded by Embretson (1983) addressing the multidimensionality that underlies intelligence tasks, thus obtaining more detailed individual data.

Despite the promising implications of this paradigm shift, only a few instruments have been developed under this approach. We believe that the issue lies not in the approach itself but rather in the limited development of psychometric models that realistically reproduce the inherent complexity of real data. We demonstrate, through an example from an analogical reasoning test, that the observed problems regarding the fit and small scope of interpretation can be overcome by selecting an appropriate analysis model, free from the limitations of the basic models developed more than two decades ago, like the linear logistic test models (Fischer 1973) and the multicomponent linear test models (Embretson and Yang 2013).

### 1.1. Analogical Reasoning

Analogical reasoning refers to the ability to identify a common relational system between two situations, exemplars, or domains and use this information to solve problems, make decisions, and generate new ideas (Gentner and Smith 2012).

This ability, distinctive to humans (Mitchell 2021), but also potentially within the capabilities of artificial systems (Combs et al. 2023), is considered a valuable resource for learning, encoding, and organizing new knowledge. In psychology, studies of analogical reasoning range from theories of intelligence to the investigation of metaphors and their uses (Ramírez et al. 2021).

The relationship between analogical reasoning and intelligence has been a significant focus in cognitive psychology research for decades. Numerous researchers posit that analogical reasoning serves as a crucial predictor of general intelligence (Cattell 1971; Holyoak 2005; Mitchell 2021; Nelson and Gillespie 1991; Spearman 1904; Sternberg 1977, 2020). Consequently, analogical reasoning tests are frequently employed as surrogate measures of general intelligence. Illustrative tests include Raven’s Progressive Matrices (Raven and Court 2003), the Abstract Reasoning Test (Embretson 1999, 2002), and Sandia Matrices (Harris et al. 2020).

**Figure 1:**
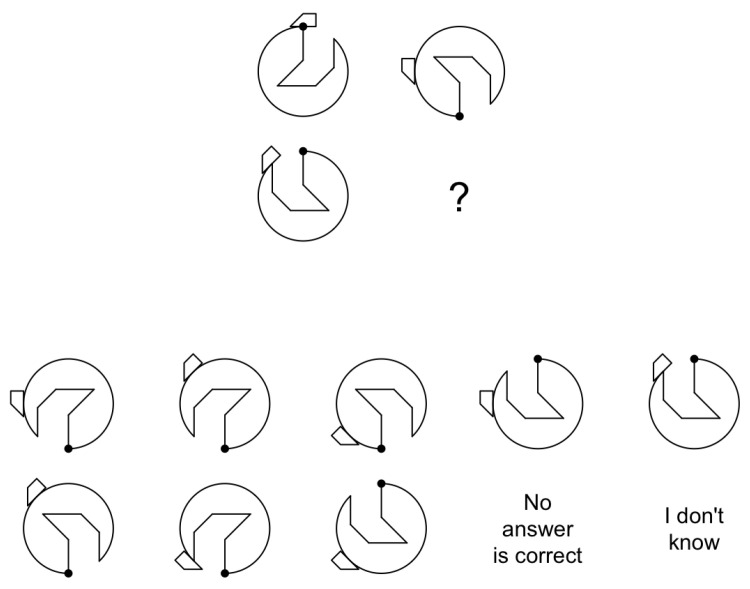
Item created using Imak R package with varied characteristics; identifying correct response requires recognizing governing pattern.

It is common for analogical reasoning tasks to utilize figurative stimuli (Blum et al. 2016; Harris et al. 2020), where items are designed based on a set of features that combine to yield varying levels of item difficulty (Arendasy and Sommer 2005). As an illustrative example, consider the item presented in Figure 1. In this item, the examinee’s goal is to discern common patterns among the elements comprising the figures and to make inferences or derive new knowledge from them (Holyoak 2005; Ruiz and Luciano 2011). To identify the correct response, one must recognize the pattern governing the sequence. During this task, examinees are tasked with identifying the missing element by focusing on the perceptual organization of the figures and their relationships.

The advantage of using analogical reasoning tasks in test development lies in the explicit information they provide about the cognitive processes guiding examinees’ responses. Breaking down items into different characteristics allows the identification of response processes, offering detailed insights into the cognitive activities involved in performance (Embretson 2019). This approach, more informative than traditional models neglecting item characteristics, optimizes the study of analogical reasoning and intelligence. It provides a detailed, ideographic understanding of examinees’ responses, enriching the analysis and enhancing construct validity by investigating processes, strategies, and knowledge structures (Embretson and Gorin 2001) influencing correct task responses (Messick 1995).

### 1.2. Construct Representation

Embretson (1983) and Embretson and Gorin (2001) introduced the concept of construct representation to explain and analyze the various cognitive processes involved in response, including sensory information processing, encoding perceived information in a format that can be processed and memorized, formulating a plan to complete a task, and evaluating the actions taken and response times (Embretson 2019; Embretson and Gorin 2001; Meyer 2010; Molenaar and Boeck 2018). This concept arose from the understanding that intelligence is a multidimensional phenomenon where multiple cognitive processes interact (Embretson 1996; Embretson and Yang 2006; Schneider and McGrew 2018; Sternberg 2018, 2020). This framework has significantly contributed to understanding the skills and strategies involved in task resolution and has enhanced the comprehension of numerous constructs in psychometrics, thereby expanding the perspective of construct validity, which was previously a less developed area in this field.

Embretson (1998) previously highlighted the advantages of constructing tests from a response process perspective. An illustrative study on the correlation between response processes and analogical reasoning is presented by Blum et al. (2016). In this study, items were automatically generated using figurative stimuli, as depicted in Figure 1. Participants were tasked with identifying a missing stimulus by discerning patterns in previously observed stimuli. The test comprised 27 items generated with the ‘IMak’ package (Blum and Holling 2018), creating figurative analogies based on five characteristics that require distinct cognitive operations: (1) rotation of the main figure, revolving around an axis in any direction by specific degrees (e.g., 90°, 180°), with a focus on identifying the axis and degree of rotation; (2) trapezoid rotation, revolving a trapezoid, emphasizing orientation changes; (3) whole figure reflection, creating a mirror image; (4) line subtraction, removing lines and altering the shape or structure, with an emphasis on identifying subtracted lines; and (5) point movement, shifting points within or between figures in a specific direction, pattern, or distance, altering appearance or structure. Each rule offers a distinct way in which objects or elements within a problem can change, challenging the solver to identify underlying patterns. The test was administered to a sample of 422 students and analyzed using the LLTM (Fischer 1973), estimating item difficulty through a linear combination of the difficulty of item characteristics.

Key findings from Blum et al. (2016) indicate that the item construction approach facilitates identifying rules significantly contributing to task difficulty. For instance, the observed movements of the main figure are strongly associated with higher difficulty levels and offer valuable insights for implementing the “construct representation” approach. In other words, this study enhances understanding of the substantive phenomenon of intelligence by indicating rules linked to certain probabilities of success.

Nevertheless, analyses and results from tests of this nature could be more comprehensive. Although the LLTM provides insights into the response processes guiding problem solving, indicating the difficulty associated with each rule, it has limitations in capturing various aspects of items and examinees due to its simplicity and lack of flexibility.

Therefore, it is imperative to develop models capable of comprehending intricate item features and addressing the true multidimensionality of data derived from such tests. This approach would yield results enabling a more thorough analysis of response processes.

### 1.3. Limitations of the LLTM and Alternative Models

In the context of the LLTM, it is crucial to acknowledge that item difficulty is intricately disassembled into individual feature difficulties. Within this framework, we denote *J* as the number of items, each uniquely indexed by the variable *j*, and *K* as the number of distinct item features, which are each distinguished by the index *k*. The cornerstone for representing this model is the **Q** matrix, structured as *J* × *K*, where a ‘1’ at the intersection of row *j* and column *k* signifies that item *j* incorporates feature *k*, while ‘0’ indicates its absence. To illustrate this concept concretely, let us examine the **Q** matrix in a specific scenario where *J* equals 5 and *K* equals 3:

$$Q_{5\times 3}=\left(\begin{array}{ccc} 1 & 1 & 1\\ 1 & 0 & 0\\ 1 & 0 & 1\\ 0 & 1 & 1\\ 1 & 0 & 0 \end{array}\right)$$

In this case, item 1 is composed by all the three features, item 2 is composed by only feature 1, item 3 is composed by features 1 and 3, and so on.

Let η be the *K*-parameter vector associated with each feature such that the vector of item difficulties is parameterized as β=Qη. Following the example, $\eta ={\left(\eta_{1},\eta_{2},\eta_{3}\right)}^{T}$. In view of the characteristics outlined above, in its most fundamental form, the LLTM posits that the probability of an examinee *i* providing a correct response to an item *j* is as follows:

(1) $$\begin{array}{cc} P(y_{ij}=1|\theta_{i},\eta ) & =\frac{\exp(\theta_{i}-\sum_{k=1}^{K}q_{jk}\eta_{k})}{1+\exp(\theta_{i}-\sum_{k=1}^{K}q_{jk}\eta_{k})} \end{array}$$

where $\theta_{i}$ is the ability of the examinee *i*.

Applied research underscores the limitations of the LLTM, primarily its restriction to estimating multidimensional models, potentially overlooking relevant information associated with response processes. Additionally, the LLTM shares several constraints with the Rasch model. Firstly, it assumes a constant discrimination parameter, fixed at 1, for each item (Fischer 1973, 1997; Fischer and Formann 1982), implying that all items provide an equal level of information. Secondly, the LLTM lacks guessing parameters, preventing the evaluation of biases that may arise in forced-choice item formats.

To address some of these limitations and estimate distinct discrimination parameters for each item, Daniel and Embretson (2010) introduced the Constrained Two-Parameter Model (2PL). His model demonstrated greater efficiency than the traditional LLTM in the analysis of mathematical reasoning tests. According to this model, the probability of successfully scoring an item *j* is

(2) $$\begin{array}{cc} P(y_{ij}=1\vee \theta_{i},\eta ,\alpha_{j}) & =\frac{\exp(\alpha_{j}\left(\theta_{i}-\sum_{k=1}^{K}q_{jk}\eta_{k}\right))}{1+\exp(\alpha_{j}\left(\theta_{i}-\sum_{k=1}^{K}q_{jk}\eta_{k}\right))} \end{array}$$

This model introduced an additional parameter, α which estimates the discrimination of each item *j*. The inclusion of this parameter represents a significant improvement, as it provides insights into which items more accurately measure the true trait level. However, it does not address the lack of the guessing parameter or offer a method for estimating unidimensional data. This aspect can be particularly relevant when dealing with data from analogical reasoning questionnaires for the reasons we will elaborate below.

### 1.4. Multicomponent Models

In the analysis of tasks based on figurative stimuli, common in analogical reasoning studies, a crucial aspect is often overlooked: the influence of physical or perceptual characteristics on the difficulty of elements. Manipulating these features can alter the perceptual elements of items, posing challenges for participants (Arendasy and Sommer 2005). This is because specific rules of these figurative elements interact in a way that significantly affects or conditions the perception of items.

When observing Figure 1, the global and local characteristics that comprise it are easily distinguishable. While global features such as orientation and circumference are essential for identifying the item as a whole, smaller features like lines and dots are not critical for this purpose Chechlacz et al. (2015). Studies on visual attention have shown that when figures are more clustered, identifying details becomes more challenging due to the competition between the global perception of the figure and the processing of details (Alvarado et al. 1999; Bundesen et al. 2015; Logan 1996). These studies explain why combinations of modifications, such as rotations of the main figure or movement of points, alter item difficulty. Based on this principle, we assume that estimated parameters can vary not only due to item feature rules (rotations, subtractions, etc.) but also due to modifications of local and global characteristics. At this point, we can hypothesize the existence of two different traits, namely the ability to detect local stimuli and the ability to detect global stimuli.

From this perspective, it is more coherent to consider a multicomponent model in which each component represents an underlying feature or specific ability measured in a set of items. One such model is the Multicomponent Latent Trait Model for Diagnosis (MLTM-D) (Embretson and Yang 2013), which is defined by its authors as a confirmatory multidimensional latent trait model. This model specifies a non-compensatory relationship between dimensions and is hierarchically organized, providing diagnosis at two levels: the component level and the skill/attribute level within the components (Embretson 2015). In this context, we argue that analogical reasoning tests using figurative stimuli, along with many other intelligence tests, could be more comprehensively explained through the MLTM-D. This contrasts with the LLTM, which does not consider the possibility of cognitive operations grouping into different dimensions or higher-order structures.

In this model, there exist M latent traits or components, which are accompanied by *m* distinct feature matrices denoted with a subscript *m* and labeled as $Q_{m}$. Under this framework, the $J_{m}$ items can be dissected into their corresponding $K_{m}$ features, which are intricately linked to a specific component denoted as m. Consequently, there emerge M distinct parameter vectors, identified as $\eta_{m}$ and associated with each $Q_{m}$. According to MLTM-D^1^, the probability of examinee *i* providing a correct response to an item *j* is as follows:

(3) $$\begin{array}{cc} P(y_{ij}=1|\theta_{i},\eta ) & =\prod_{m=1}^{M}{\left(\frac{\exp(\theta_{im}-\sum_{k=1}^{K}q_{mjk}\eta_{mk})}{1+\exp(\theta_{im}-\sum_{k=1}^{K}q_{mjk}\eta_{mk})}\right)}^{C_{jm}} \end{array}$$

where C is a J × M component matrix with entry 1 in row *j* and column m if a feature which is present in item *j* is related to the trait $\theta_{m}$ and 0 otherwise.

The idea behind the MLTM-D is that an examinee *i* can master component m with a given probability so that the probabilities of mastering the *M* components required to solve an item combine multiplicatively to yield the probability of correctly answering it. This model is non-compensatory because the presence of features in an item that are not related to a component *m* does not influence the probability of succeeding such an item.

For simplification, all the feature matrices $Q_{m}$ in the MLTM-D may be represented in a single feature matrix Q. Let J and K become again the total number items and item features in the test, respectively. Let Q be now a block diagonal matrix containing all the individual $Q_{m}$ matrices. For example, let *J* = 10, *K* = 6 and *M* = 2, and consider the following Q matrix,

$$Q_{10\times 6}=\left(\begin{array}{cc} Q_{1_{5\times 3}} & 0\\ 0 & Q_{2_{5\times 3}} \end{array}\right)=\left(\begin{array}{c} \begin{array}{cc} \overset{\mathrm{Features}\;\mathrm{for}\;\mathrm{component}\;1}{\overbrace{\begin{array}{ccc} 1 & 1 & 1\\ 1 & 0 & 0\\ 1 & 0 & 1\\ 0 & 1 & 1\\ 0 & 1 & 0\\ 0 & 0 & 0\\ 0 & 0 & 0\\ 0 & 0 & 0\\ 0 & 0 & 0\\ 0 & 0 & 0 \end{array}}} & \overset{\mathrm{Features}\;\mathrm{for}\;\mathrm{component}\;2}{\overbrace{\begin{array}{ccc} 0 & 0 & 0\\ 0 & 0 & 0\\ 0 & 0 & 0\\ 0 & 0 & 0\\ 0 & 0 & 0\\ 1 & 0 & 1\\ 1 & 0 & 0\\ 0 & 1 & 1\\ 0 & 0 & 1\\ 1 & 1 & 0 \end{array}}} \end{array} \end{array}\right)$$

Within this alternative representation, η must be a matrix of dimensions *K × M* with the following parameters:

$$\eta_{6\times 2}=\left(\begin{array}{cc} \eta_{11} & 0\\ \eta_{21} & 0\\ \eta_{31} & 0\\ 0 & \eta_{42}\\ 0 & \eta_{52}\\ 0 & \eta_{62} \end{array}\right)$$

Finally, because the five first items are related to the first component and the last five are related to the second component, the component matrix associated to Q is

$$C_{10\times 2}=\left(\begin{array}{cc} 1 & 0\\ 1 & 0\\ 1 & 0\\ 1 & 0\\ 1 & 0\\ 0 & 1\\ 0 & 1\\ 0 & 1\\ 0 & 1\\ 0 & 1 \end{array}\right)$$

In real applications, items may be composed of features related to different components. That is, items may cross-load in more than one $\theta_{m}$. Hence, representing the MLTM-D with a single Q matrix may be helpful to visualize such cross-loadings. Consider the same Q matrix as before but with item 1 containing one feature from component 2:

$$Q_{10\times 6}=\left(\begin{array}{cc} \overset{\mathrm{Features}\;\mathrm{for}\;\mathrm{component}\;1}{\overbrace{\begin{array}{ccc} 1 & 1 & 1\\ 1 & 0 & 0\\ 1 & 0 & 1\\ 0 & 1 & 1\\ 0 & 1 & 0\\ 0 & 0 & 0\\ 0 & 0 & 0\\ 0 & 0 & 0\\ 0 & 0 & 0\\ 0 & 0 & 0 \end{array}}} & \overset{\mathrm{Features}\;\mathrm{for}\;\mathrm{component}\;2}{\overbrace{\begin{array}{ccc} 1 & 0 & 0\\ 0 & 0 & 0\\ 0 & 0 & 0\\ 0 & 0 & 0\\ 0 & 0 & 0\\ 1 & 0 & 1\\ 1 & 0 & 0\\ 0 & 1 & 1\\ 0 & 0 & 1\\ 1 & 1 & 0 \end{array}}} \end{array}\right)\;C_{10\times 2}=\left(\begin{array}{cc} 1 & 1\\ 1 & 0\\ 1 & 0\\ 1 & 0\\ 1 & 0\\ 0 & 1\\ 0 & 1\\ 0 & 1\\ 0 & 1\\ 0 & 1 \end{array}\right)$$

In this case, the first row of the C matrix must specify that both components are required to solve the item. On the other hand, the η matrix remains the same as before. There is no $\eta_{41}$ parameter to be estimated for component 1 and feature 4, because this feature is not related to component 1. Therefore, the nature of the MLTM-D is non-compensatory.

Notwithstanding, if feature 4 was also related to component 1, then we would have to estimate the parameter $\eta_{41}$ and the C matrix would become

$$C_{10\times 2}=\left(\begin{array}{cc} 1 & 1\\ 1 & 0\\ 1 & 0\\ 1 & 0\\ 1 & 0\\ 1 & 1\\ 1 & 1\\ 0 & 1\\ 0 & 1\\ 1 & 1 \end{array}\right)$$

That is, component 1 is now also involved in the solution to items 6, 7 and 10. It is noteworthy that in instances where M equals 1, the MLTM-D effectively reduces to the LLTM.

Given that we have delved into the key features of the psychometric model proposed by Embretson and Yang (2013), we now present an illustration applying Embretson’s concepts and contributing our theory on analogical reasoning. To do so, we draw on insights derived from the research conducted by Blum and colleagues (Blum et al. 2016). Returning to their studies, we carefully selected four items from their dataset to demonstrate how the concepts under investigation can be applied within a multicomponent model.

**Figure 2:**
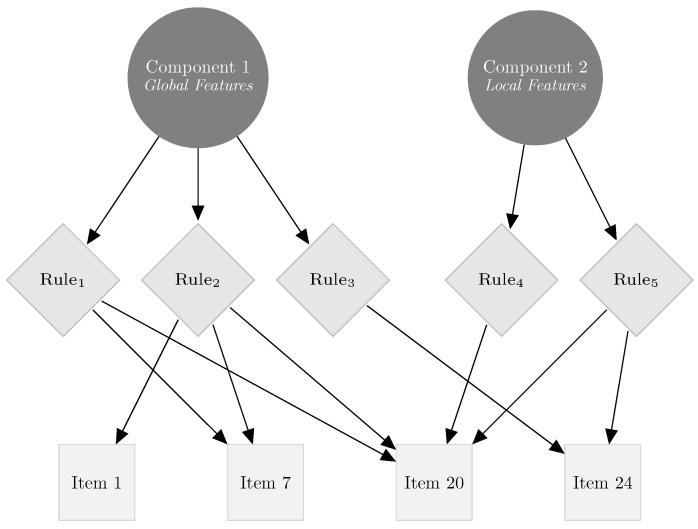
Hierarchical structure of a multicomponent test: rules and components. This diagram depicts a hierarchical model of a figurative stimulus analysis test, featuring two main components with specific rules. At a tertiary level, it illustrates four items from the Blum test, which are each composed of specific rule combinations. For instance, item 24 is influenced by rules 3 and 5.

Figure 2 visually encapsulates this application, presenting the five distinct rule categories outlined by Blum. For instance, when examining item 7, the probability of a correct response is intricately tied to rules 1 and 2. This necessitates the examinee’s ability to discern alterations in the overall structure of the figures to successfully answer the item. In contrast, item 20, influenced by rules 4 and 5, demands proficiency in recognizing local differences between figures. Notably, this item encompasses rules 1, 2, 4, and 5, with the likelihood of a correct response influenced by both these specific rules and the two overarching components that encapsulate them.

From the perspective of response processes, we can conclude that multicomponent models offer considerably more detailed and comprehensive information compared to traditional models.

So far, our main objective has been to emphasize the significant limitations of the LLTM model in modeling this type of data while underscoring the superior coherence of multicomponent models. However, our focus has not been solely on seeking a coherent model; we have also explored the relationship of these models to response processes. In this context, a clear weakness of the MLTM-D, as evidenced in its mathematical formulation, is the lack of information regarding item discrimination and other relevant parameters such as guessing. Although Embretson (2015) incorporates a discrimination parameter, it addresses discrimination for each component, assuming that the relationship between the probability of a correct response and component *m* follows the same logistic form for all items. Therefore, to overcome these limitations, we propose a Bayesian generalization of the MLTM-D to a three-parameter version (3PL).

We hypothesize that this generalization will yield better results in prediction and fit than its predecessors. However, our interest is not solely focused on fit but rather on the explanatory power of data with heterogeneous characteristics. Our dissertation stems from the problem that previous models might present fit issues precisely because their constraints could omit relevant aspects of the data. Therefore, this initiative aims to improve the evaluation and diagnosis of analogical reasoning questionnaires and, more broadly, the assessment of intelligence.

### 1.5. The Generalized Multicomponent Latent Trait Model for Diagnosis (GMLTM-D)

The MLTM-D surpasses the unidimensional limitation of the LLTM yet still offers room for improvement by relaxing some constraints. First, allowing the discrimination of each item within each component to differ enables the variation of the relationship between a latent component and the probability of success across items. Second, guessing parameters may be introduced in the model specification. Our generalized model is presented as follows:

(4) $$P\left(y_{ijm}=1|\theta_{im},\eta_{mk},\alpha_{jm},c_{j}\right)=\frac{\exp(\alpha_{jm}\left(\theta_{im}-\sum_{k=1}^{K}q_{mjk}\eta_{mk}\right))}{1+\exp(\alpha_{jm}\left(\theta_{im}-\sum_{k=1}^{K}q_{mjk}\eta_{mk}\right))}\;$$

(5) $$P\left(y_{ij}=1|c_{j}\right)=c_{j}+\left(1-c_{j}\right)\prod_{m=1}^{M}P{\left(y_{ijm}\right)}^{C_{jm}}\;$$

The equation of model (4) is an extension of a two-parameter logistic model of IRT for mastering each component. Then, the joint probability of success on the item is related to the product of the probabilities associated with mastering each component (see Equation (5)), in the sense of a three-parameter model.

This model constitutes a generalization of the MLTM-D, encompassing additional parameters commonly encountered in other IRT models, such as discrimination ($\alpha_{jm}$) and guessing ($c_{j}$). While the guessing parameter varies from one item to another, discrimination parameters may be identical for multiple items. Within the GMLTM-D framework, items composed of the same feature combinations within the same component maintain consistent discriminative power. In essence, discrimination is conceptualized as an inherent property of the feature combinations or rules that constitute the item. This perspective aligns with the fundamental notion that rules serve as the elementary units of items. If two items share the same rules, both their difficulty and their relationship to the skill level are comparable.

On the contrary, the guessing parameters, denoted as $c_{j}$, account for correct item responses when the examinee does not master the rules comprising the item. For instance, the examinee may not fully grasp the relationship between item characteristics, complicating the selection of the correct answer due to the presence of multiple options that share similarities. In such cases, they might discard less plausible options and make a random selection of what they deem correct. Consequently, their response would incorporate a guessing component. The inclusion of this parameter in the model aims to capture variability in responses that is not solely contingent on understanding the item’s characteristics. This stochastic element reflects the possibility that on certain occasions, the choice of the correct answer may be due to guessing, considering that some items may contain higher-quality distractors than others.

### 1.6. Comparison of Bayesian Models in Data Analysis

In this study, an additional analysis was conducted on the data presented by Blum et al. (2016) using Bayesian versions of the LLTM, MLTM-D, and GMLTM-D models. Our hypothesis is that the GMLTM-D will exhibit superior predictive performance, better cross-validation results, and greater parsimonious fit compared to the other competing models.

## 2. Methods

We conducted a reanalysis of the data from Blum et al. (2016) using the Bayesian versions of the LLTM, MLTM-D and GMLTM-D models. We assessed responses from 383 participants using the 27 items from the original test. The data and code for these models are accessible via open access at the following link: https://bit.ly/3xrP3Rq (accessed on 17 July 2024).

The item features used to build the Q matrix remained consistent and included rotation of the main figure, trapezium rotation, reflection of the main shape, subtraction of lines, and dot movement. The complete Q matrix is detailed in Table 1. These analyses were conducted after simulation studies aimed at verifying the parameter recovery of the GMLTM-D, an example of which can be seen in Appendix C.

In the MLTM-D and GMLTM-D models, the *Q* matrix differentiated between global features, such as the rotation of the main figure, trapezium rotation, and reflection of the main shape, and local features, such as line subtraction and point movement. Consequently, parameter estimation was performed with two components, assigning rules 1, 2, and 3 to component 1, and rules 4 and 5 to component 2. This aimed to compare multidimensional models with LLTM, showing GMLTM-D’s enhanced ability to capture data nuances, which is often obscured by less flexible models.

In the GMLTM-D, items sharing the same combinations of features within the same component remained equally discriminative. Items composed by the same feature combinations within the same component should remain equally discriminative because discrimination is an item property and, hence, a property of the feature combinations that make up the item.

The Bayesian model estimation was performed using Hamiltonian Monte Carlo sampling, implemented in the GMLTM package, which is built upon the R CmdStan package. The GMLTM package can be accessed on GitHub: https://github.com/Marcosjnez/GMLTM (accessed on 17 July 2024). Model validation was conducted following the recommendations of Gelman et al. (2020).

To assess the adequacy of the prior distributions, pre-emptive predictive checks were conducted to verify the coherence of the model’s prior distributions, as detailed graphically in the Appendix A.

This was completed to ensure the credibility of the generated data and to prevent any bias toward incoherent patterns. Following the model estimation, a verification process was conducted to evaluate chain mixing and identify divergences.

The anticipated distributions were tailored in accordance with the following patterns:

$$\theta_{im}∼\mathrm{Normal}\left(0,1\right),\;\eta_{mk}∼\mathrm{Normal}\left(0,1\right),\;\alpha_{jm}∼\mathrm{Normal}\left(0,1\right),\;\alpha_{jm}\geq 0,\;c_{j}∼\mathrm{Beta}(3,20).$$

Subsequently, posterior predictive checks were conducted to assess model performance. One method employed to evaluate performance involved assessing the bias between the Posterior Probability Intervals and the observed data. Additionally, the Standardized Root Mean Square Residual (SRMR) was calculated to evaluate the three models. Furthermore, the Widely Applicable Information Criterion (WAIC) (Watanabe 2013) was also used for model comparison. It also provides information on overfitting, as it includes a penalty for model complexity, suggesting the model with the best balance between fit and complexity. WAIC serves as a metric for evaluating the goodness of fit of a statistical model to the observed data, considering both model parsimony and predictive accuracy. A lower WAIC value indicates a better model fit in terms of parsimony and superior predictive capacity compared to a model with a higher WAIC value (Gelman et al. 2013; Vehtari et al. 2017).

## 3. Results

In the initial analysis, the GMLT-D model demonstrated exceptional performance in capturing the marginal proportions of correct responses derived from the predictive posterior distribution of items. Table 2 clearly illustrates that the GMLT-D model provided the most accurate predictions through its expected a posteriori (EAP) estimates and corresponding credibility intervals. This model exhibited only 15% of predictions that did not align with the observed data. In contrast, the MLTM-D model displayed a 67% rate of discordant predictions, while the LLTM model revealed an 81% inconsistency with the observed data. In terms of the biases between the Posterior Probability Intervals and the observed data, they were lower in the GMLTM-D, which exhibited a bias of 0.009, while the MLTM-D showed a bias of 0.231, and the LLTM revealed a bias of 0.385. Additionally, the results indicated that the GMLT-D model achieved the lowest level with an SRMR of 0.02, followed by the MLTM-D with an SRMR of 0.09, and the LLTM with an SRMR of 0.10.

**Figure 3:**
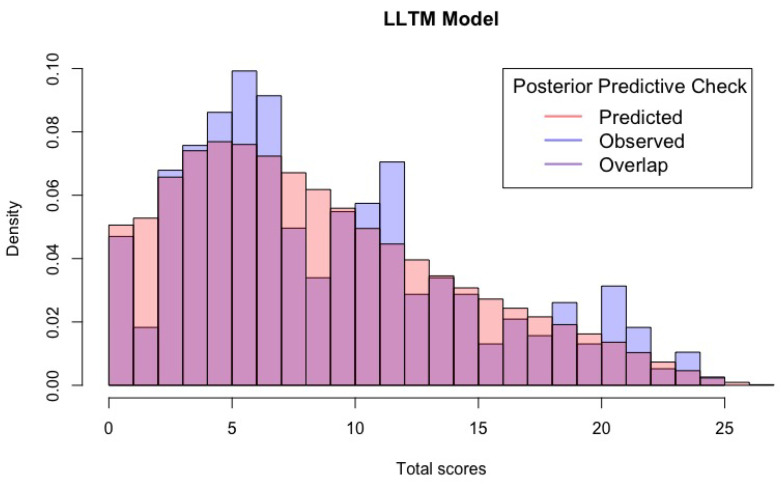

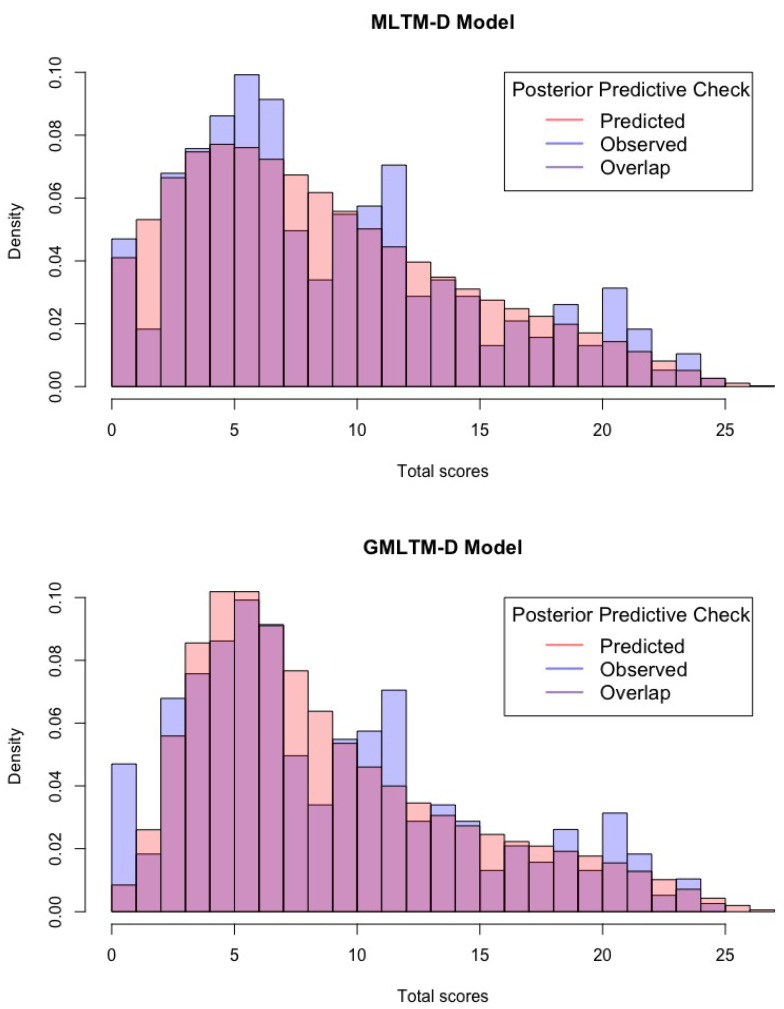
Posterior predictive check histograms. These histograms compare predicted and observed distributions, enabling a visual assessment of model fit.

Figure 3 provides a visual representation of the precise alignment between the model’s predictions and the empirical observations after conducting comprehensive posterior predictive checks with the exception of cases where examinees exhibited lower response levels. In contrast, both the MLTM-D and LLTM models presented predictions that deviated from the credibility interval concerning marginal proportions of correct responses. These findings are substantiated by the results presented in Table 2.

When evaluating the fit of the models, it is noteworthy to mention that the WAIC score was lower in GMLT-M, with a WAIC of 10,317.42, in contrast to the WAIC of 10,758.56 for MLTM-D and 10,777.46 for LLTM. This signifies a superior predictive capability and fit in GMLTM-D compared to other models.

The findings presented in Table 3 reveal the difficulties associated with each rule. In this context, rule 5, known as “point movement”, stands out by displaying negative scores, positioning it as the rule with the least difficulty. Its score suggests significant facilitation in item resolution. In contrast, rule 1, “Principal Figure Rotation”, along with rule 3, “Entire Figure Reflection”, exhibit moderate difficulties. On the other hand, rules 2, “Trapezium Rotation”, and 4, “Line Subtraction”, emerge as the most challenging. Notably, the simplest and most challenging rules are found within the local component, while rules with moderate and high difficulties reside within the global component.

Additionally, Table 4 provides estimates for the 27 items of the analogical reasoning test using the GMLT-D model. It is noteworthy that items composed of rules from the local component exhibit lower difficulty compared to items from the global component, especially when rule 4 from the local component is not included, as evidenced in Table 3, which has proven to be the most challenging. In this context, the rules from the global component contribute the most difficulty to the items, as their three rules show moderate and high difficulty levels. In contrast, the local component contributes with a slightly higher discriminative power.

Moreover, Table 4 reveals that items incorporating two rules from the global component and two from the local component show significantly higher levels of difficulty and discrimination along with lower guessing rates. This pattern is evident in item 11, highlighted in bold, with parameters of b = 1.16 for component 1 and 0.08 for component 2, and a = 1.66 for component 1 and 1.78 for component 2, along with a probability of random guessing of 8%. In contrast, items involving the local component rule “point movement” and not including the “line subtraction” rule from the local component exhibit notably low difficulty parameters. Item 8, also highlighted in bold, illustrates this point with b = 1.16 for component 1 and −0.79 for component 2, and a discrimination of a = 1.66 for component 1 and 2.66 for component 2. These findings support the facilitating contribution of the local component to task performance. It is noteworthy that the guessing parameter consistently remains low across all items, except for item 2, where the probability of guessing at low skill levels reaches 57%.

**Figure 4:**
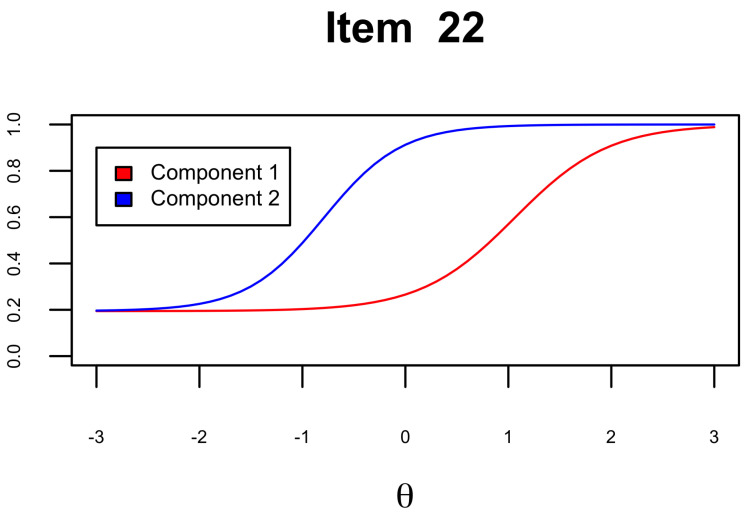
Item characteristic curve. The figure depicts item characteristic curves, each linked to the two components, illustrating how success probability is affected by individual contributions.

The characteristic curve presented in Figure 4 illustrates the curves of the two components that make up the item. As evidenced in this figure, especially in relation to item 22 and supported by the information in Table 4, the difficulty in obtaining accuracy is more affected by the contribution of component 1. This pattern is consistently observed in other characteristic curves available in the Appendix B.

## 4. Discussion

This study aimed to redefine the underlying logic in psychometric explanations of constructs such as intelligence, with a specific focus on the relationship between analogical reasoning and construct representation studies. Investigating the processes involved in cognitive task resolution is deemed essential for constructing validity evidence (Messick 1995). Enhancing the study of analogical reasoning allows for more precise conclusions about general intelligence. Our central objective was to identify a psychometric model enabling the analysis of the heterogeneous characteristics of analogical reasoning tests, thereby offering insights into response processes.

Analyzing commonly used psychometric models for analogical reasoning test data, such as the LLTM, revealed certain limitations. Firstly, these models are restrictive and fail to adequately fit many scenarios (Baghaei and Hohensinn 2017; Fischer and Formann 1982). Moreover, they may conflate variability provided by item characteristics or simply do not allow analysis when data are grouped into multiple components. In response to these limitations, proposals for multicomponent models have emerged.

The choice of multicomponent models is grounded in the notion that the physical characteristics of items, represented in the Q-matrix, may give rise to distinct components. For instance, when analyzing Raven matrices (Raven and Court 2003) or items created using the Imak package (Blum and Holling 2018), it becomes evident that physical differences in the figures can be categorized into different dimensions, such as global and local features. We argue that this distinction is crucial not only in terms of response processes but also for analyzing and isolating perceptual characteristics that may influence measurement.

Furthermore, we posit that multicomponent models are justified as there are skills dedicated to solving specific problems or rules that may be nested within different components. This allows us to obtain information about the domain of a group of rules or nested cognitive operations within a component, which is a type of information that is limited in classical models like the LLTM.

Embretson and Yang (2013)’s multidimensional proposal represents a significant advancement in this field. However, there are aspects that still warrant exploration to enhance our understanding of the data. Our model emerges as a response to this challenge. Firstly, it allows for the estimation of multiple components, following the structure of the predecessor model, while also estimating the parameters of IRT: discrimination, difficulty, and guessing. These parameters, in addition to informing about the metric properties of tasks, aid in comprehending response processes by revealing which characteristics hinder or discriminate better the examinees’ skill level. This novelty is implemented with component-wise estimation, providing us with parameter information for both features nested in component 1 and component 2. Precisely, this enables us to analyze response processes and achieve more accurate conclusions about examinees’ trait level.

As reflected in this study, our approach arises from the need to propose more flexible models to better represent the underlying processes involved in analogical reasoning tests and potentially other types of measurements that inherently possess heterogeneous data. We have explained why, in this case, the theoretical choice of these models makes more sense to achieve a more detailed explanation of the data, which is something that is not entirely possible with more restrictive models. For this reason, we consider that the parametric complexity of our generalized model is justified.

To test our assumptions, we reanalyzed the results of Blum’s items (Blum et al. 2016), which were originally studied using the LLTM. Our findings indicated that the proposed model, GMLTM-D, surpasses the LLTM and MLTM-D models in terms of cross-validation, demonstrating no overfitting despite having more parameters. Additionally, the GMLTM-D exhibits greater predictive accuracy and a superior ability to precisely capture the marginal proportions of correct responses derived from the posterior predictive distribution of items.

Examining the estimates of rule difficulties and inherent IRT parameters in our model, we observed that the difficulty was more pronounced for rules nested in the global component. However, the rule of line subtraction, corresponding to the local component, proved to be the most challenging. An intriguing finding is that the points movement rule incorporates indicators that facilitate the correct response to items. In general, items composed of rules from the local component are less difficult compared to those from the global component. Furthermore, in this reanalysis, we noted that the majority of items exhibited high levels of discrimination. Additionally, significant levels of guessing were observed in only one of the items.

Some of these results, such as the greater difficulty of rules like the movement of the main figure, which in our case was grouped in the global component, align with previous analyses by Blum et al. (2016). However, our specific contribution to the field of figural analogical reasoning lies in being able to showcase, firstly, the richness of analyses from a multicomponent perspective, and more specifically, by adopting an approach that highlights cognitive operations that increase the probability of success for examinees. For example, we believe that in tasks of this nature, examinees can detect changes from Figure A to B by observing more local details of the figure, specifically in this case, the movement of points, providing them with clues to succeed in figural analogies. However, changes at a global level are less detectable and require a higher level of analogical reasoning from the examinee to be successfully resolved.

In conclusion, we have presented a discussion on handling data with heterogeneous characteristics that are more amenable to analysis through multicomponent models. Our implementation provides a solution that exceeds the constraints of traditional approaches and focuses more on detailed analyses of individual characteristics and response processes. We propose that future implementations of the GMLTM-D could explore interactions between rules. By exploring the joint influence of these rules, the information provided by the model could be enriched, aiding in the establishment of a psychometric criterion supporting rule combination. This enhancement would contribute to substantive advances in item or cognitive task design through empirical arguments, as argued by Embretson (1998).

Regarding the limitations of this study, it should be noted that our model was not accurate in predicting when examinees had few correct responses on the test, and the marginal proportions of correct responses showed predictions outside the confidence range for three items. These limitations suggest that future implementations must be carried out with strict control and care, as recommended by Gelman et al. (2020).

## 5. Conclusions

In explaining data heterogeneity in cognitive performance tests, traditional models face challenges. Hence, there is a need for more flexible models like GMLTM-D, which tackle data from multidimensional perspectives aligned with current understanding of intelligence and cognitive performance. Through such approaches, solutions can be proposed to enhance comprehension not only of data in cognitive reasoning tests but also of examinees’ response processes. This, in turn, could broaden ideographic understandings of intelligence and contribute to a greater validity of measures. 

## Figures and Tables

**Table 1:** Q Matrix.

Item	Rules				
	Rule 1	Rule 2	Rule 3	Rule 4	Rule 5
1	0	1	0	0	0
2	0	0	1	0	0
3	1	0	0	0	0
4	0	0	0	0	1
5	1	0	0	0	0
6	0	1	0	0	0
7	1	1	0	0	0
8	0	1	1	0	1
9	1	1	0	1	1
10	1	1	0	0	0
11	0	1	1	1	1
12	1	0	0	0	1
13	1	1	0	0	1
14	1	1	0	1	1
15	0	1	0	0	1
16	1	1	0	0	1
17	1	1	0	1	1
18	1	1	0	0	0
19	0	1	1	0	1
20	1	1	0	1	1
21	0	1	1	0	0
22	1	1	0	0	1
23	0	1	1	1	1
24	0	0	1	0	1
25	1	1	0	0	1
26	0	1	1	0	0
27	1	1	0	0	1

Rule 1: principal figure rotation; Rule 2: trapezium rotation; Rule 3: entire figure reflection Rule 4: line subtraction; Rule 5: point movement.

**Table 2:** Marginal success rates for the three estimated models.

Item	GMLT-D					MLTM-D					LLTM			
	2.5%	EAP	97.5%	Dobs		2.5%	EAP	97.5%	Dobs		2.5%	EAP	97.5%	Dobs
1	0.47	0.51	0.56	0.52		0.39	0.42	0.44	**0.52**		0.39	0.41	0.43	**0.52**
2	0.70	0.75	0.79	0.78		0.50	0.52	0.55	**0.78**		0.47	0.49	0.51	**0.78**
3	0.45	0.48	0.52	0.48		0.47	0.49	0.51	0.48		0.45	0.47	0.49	0.48
4	0.73	0.76	0.79	0.74		0.77	0.79	0.82	**0.74**		0.55	0.57	0.59	**0.74**
5	0.45	0.49	0.52	0.47		0.47	0.49	0.51	0.47		0.45	0.47	0.49	0.47
6	0.37	0.40	0.44	0.42		0.39	0.42	0.44	0.42		0.39	0.41	0.43	**0.42**
7	0.34	0.38	0.43	0.40		0.33	0.34	0.36	**0.40**		0.29	0.31	0.33	**0.40**
8	0.23	0.26	0.29	**0.22**		0.28	0.30	0.32	**0.22**		0.31	0.32	0.34	**0.22**
9	0.17	0.20	0.24	0.22		0.16	0.17	0.19	**0.22**		0.16	0.17	0.19	**0.22**
10	0.34	0.38	0.42	0.40		0.33	0.34	0.36	**0.40**		0.29	0.31	0.33	**0.40**
11	0.16	0.20	0.23	0.22		0.17	0.19	0.20	0.22		0.17	0.18	0.20	**0.22**
12	0.36	0.39	0.43	0.39		0.38	0.40	0.42	0.39		0.44	0.46	0.49	**0.39**
13	0.25	0.28	0.31	0.28		0.27	0.28	0.29	0.28		0.29	0.31	0.32	0.28
14	0.16	0.19	0.23	0.19		0.16	0.17	0.19	0.19		0.16	0.17	0.19	**0.19**
15	0.42	0.46	0.51	0.50		0.32	0.34	0.36	**0.50**		0.38	0.40	0.43	**0.50**
16	0.33	0.37	0.42	0.41		0.27	0.28	0.29	**0.41**		0.29	0.31	0.32	**0.41**
17	0.13	0.15	0.18	0.14		0.16	0.17	0.19	**0.14**		0.16	0.17	0.19	**0.14**
18	0.22	0.25	0.28	**0.20**		0.33	0.34	0.36	**0.20**		0.29	0.31	0.33	**0.20**
19	0.25	0.29	0.32	0.31		0.28	0.30	0.32	0.31		0.31	0.32	0.34	**0.31**
20	0.16	0.18	0.22	0.18		0.16	0.17	0.19	0.18		0.16	0.17	0.19	**0.18**
21	0.29	0.33	0.37	0.34		0.35	0.37	0.39	**0.34**		0.31	0.33	0.35	0.34
22	0.30	0.34	0.38	0.36		0.27	0.28	0.29	**0.36**		0.29	0.31	0.32	**0.36**
23	0.12	0.14	0.17	**0.11**		0.17	0.19	0.20	**0.11**		0.17	0.18	0.20	**0.11**
24	0.40	0.44	0.48	0.46		0.40	0.42	0.44	**0.46**		0.46	0.48	0.51	**0.46**
25	0.22	0.25	0.28	0.26		0.27	0.28	0.29	0.26		0.29	0.31	0.32	**0.26**
26	0.23	0.26	0.29	**0.20**		0.35	0.37	0.39	**0.20**		0.31	0.33	0.35	**0.20**
27	0.19	0.21	0.24	0.17		0.27	0.28	0.29	**0.17**		0.29	0.31	0.32	**0.17**

Posterior Probability Intervals (2.5–97.5%); EAP: Expected a Posteriori; Dobs: Observed data. Dobs outside the Posterior Probability Intervals are highlighted in bold.

**Table 3:** Estimation of the difficulties of the components.

	GMLT-D			MLTM-D			LLTM
Rules	Component 1	Component 2		Component 1	Component 2		Component 1
	IC [2.5%, 97.5%]	IC [2.5%, 97.5%]		IC [2.5%, 97.5%]	IC [2.5%, 97.5%]		IC [2.5%, 97.5%]
η1	0.29 [0.15, 0.43]	0.00 [0.00, 0.00]		0.32 [0.22, 0.42]	0.00 [0.00, 0.00]		0.53 [0.41, 0.64]
η2	0.77 [0.64, 0.92]	0.00 [0.00, 0.00]		0.65 [0.55, 0.75]	0.00 [0.00, 0.00]		0.83 [0.73, 0.93]
η3	0.39 [0.22, 0.56]	0.00 [0.00, 0.00]		0.20 [0.09, 0.31]	0.00 [0.00, 0.00]		0.43 [0.30, 0.55]
η4	0.00 [0.00, 0.00]	0.87 [0.48, 1.22]		0.00 [0.00, 0.00]	1.34 [1.09, 1.62]		0.91 [0.77, 1.06]
η5	0.00 [0.00, 0.00]	−0.79 [−1.01, −0.60]		0.00 [0.00, 0.00]	−1.23 [−1.48, −1.02]		0.03 [−0.07, 0.13]

Component 1: Global characteristics of the items. Component 2: Local characteristics of the items. In the LLTM, only one component is observed.

**Table 4:** IRT parameter estimates in the GMLTM-D.

	Item Discrimination Parameter (*a*)			Item Difficulty Parameter (*b*)			Item Guessing Parameter (*c*)
Item	Component 1	Component 2		Component 1	Component 2		Component 1
	EAP [2.5%, 97.5%]	EAP [2.5%, 97.5%]		EAP [2.5%, 97.5%]	EAP [2.5%, 97.5%]		EAP [2.5%, 97.5%]
1	1.42 [1.03, 1.93]	0.00 [0.00, 0.00]		0.77 [0.64, 0.92]	0.00 [0.00, 0.00]		0.27 [0.19, 0.35]
2	2.42 [1.64, 3.37]	0.00 [0.00, 0.00]		0.39 [0.22, 0.56]	0.00 [0.00, 0.00]		0.57 [0.50, 0.64]
3	1.65 [1.32, 2.02]	0.00 [0.00, 0.00]		0.29 [0.15, 0.43]	0.00 [0.00, 0.00]		0.07 [0.02, 0.14]
4	0.00 [0.00, 0.00]	2.66 [1.79, 3.67]		0.00 [0.00, 0.00]	−0.79 [−1.01, −0.60]		0.07 [0.01, 0.15]
5	1.65 [1.32, 2.02]	0.00 [0.00, 0.00]		0.29 [0.15, 0.43]	0.00 [0.00, 0.00]		0.08 [0.02, 0.15]
6	1.42 [1.03, 1.93]	0.00 [0.00, 0.00]		0.77 [0.64, 0.92]	0.00 [0.00, 0.00]		0.11 [0.04, 0.18]
7	2.19 [1.81, 2.66]	0.00 [0.00, 0.00]		1.06 [0.91, 1.21]	0.00 [0.00, 0.00]		0.20 [0.15, 0.26]
**8**	**1.66 [1.32, 2.05]**	**2.66 [1.79, 3.67]**		**1.16 [0.99, 1.33]**	**−0.79 [−1.01, −0.60]**		**0.09 [0.05, 0.14]**
9	2.19 [1.81, 2.66]	1.78 [0.94, 2.84]		1.06 [0.91, 1.21]	0.08 [−0.30, 0.40]		0.09 [0.06, 0.13]
10	2.19 [1.81, 2.66]	0.00 [0.00, 0.00]		1.06 [0.91, 1.21]	0.00 [0.00, 0.00]		0.20 [0.15, 0.26]
**11**	**1.66 [1.32, 2.05]**	**1.78 [0.94, 2.84]**		**1.16 [0.99, 1.33]**	**0.08 [−0.30, 0.40]**		**0.08 [0.05, 0.13]**
12	1.65 [1.32, 2.02]	2.66 [1.79, 3.67]		0.29 [0.15, 0.43]	−0.79 [−1.01, −0.60]		0.07 [0.03, 0.13]
13	2.19 [1.81, 2.66]	2.66 [1.79, 3.67]		1.06 [0.91, 1.21]	−0.79 [−1.01, −0.60]		0.12 [0.07, 0.16]
14	2.19 [1.81, 2.66]	1.78 [0.94, 2.84]		1.06 [0.91, 1.21]	0.08 [−0.30, 0.40]		0.08 [0.05, 0.12]
15	1.42 [1.03, 1.93]	2.66 [1.79, 3.67]		0.77 [0.64, 0.92]	−0.79 [−1.01, −0.60]		0.27 [0.20, 0.34]
16	2.19 [1.81, 2.66]	2.66 [1.79, 3.67]		1.06 [0.91, 1.21]	−0.79 [−1.01, −0.60]		0.23 [0.18, 0.29]
17	2.19 [1.81, 2.66]	1.78 [0.94, 2.84]		1.06 [0.91, 1.21]	0.08 [−0.30, 0.40]		0.04 [0.01, 0.06]
18	2.19 [1.81, 2.66]	0.00 [0.00, 0.00]		1.06 [0.91, 1.21]	0.00 [0.00, 0.00]		0.03 [0.01, 0.06]
19	1.66 [1.32, 2.05]	2.66 [1.79, 3.67]		1.16 [0.99, 1.33]	−0.79 [−1.01, −0.60]		0.12 [0.07, 0.18]
20	2.19 [1.81, 2.66]	1.78 [0.94, 2.84]		1.06 [0.91, 1.21]	0.08 [−0.30, 0.40]		0.07 [0.04, 0.11]
21	1.66 [1.32, 2.05]	0.00 [0.00, 0.00]		1.16 [0.99, 1.33]	0.00 [0.00, 0.00]		0.13 [0.07, 0.18]
22	2.19 [1.81, 2.66]	2.66 [1.79, 3.67]		1.06 [0.91, 1.21]	−0.79 [−1.01, −0.60]		0.19 [0.15, 0.25]
23	1.66 [1.32, 2.05]	1.78 [0.94, 2.84]		1.16 [0.99, 1.33]	0.08 [−0.30, 0.40]		0.02 [0.01, 0.05]
24	2.42 [1.64, 3.37]	2.66 [1.79, 3.67]		0.39 [0.22, 0.56]	−0.79 [−1.01, −0.60]		0.18 [0.11, 0.25]
25	2.19 [1.81, 2.66]	2.66 [1.79, 3.67]		1.06 [0.91, 1.21]	−0.79 [−1.01, −0.60]		0.08 [0.05, 0.13]
26	1.66 [1.32, 2.05]	0.00 [0.00, 0.00]		1.16 [0.99, 1.33]	0.00 [0.00, 0.00]		0.03 [0.01, 0.07]
27	2.19 [1.81, 2.66]	2.66 [1.79, 3.67]		1.06 [0.91, 1.21]	−0.79 [−1.01, −0.60]		0.03 [0.01, 0.06]

Component 1: Global characteristics of the items. Component 2: Local characteristics of the items. The probability of success by guessing is calculated in the two components. The IRT parameters of two items used as examples in the results explanation are highlighted in bold.

**Figure A1:**
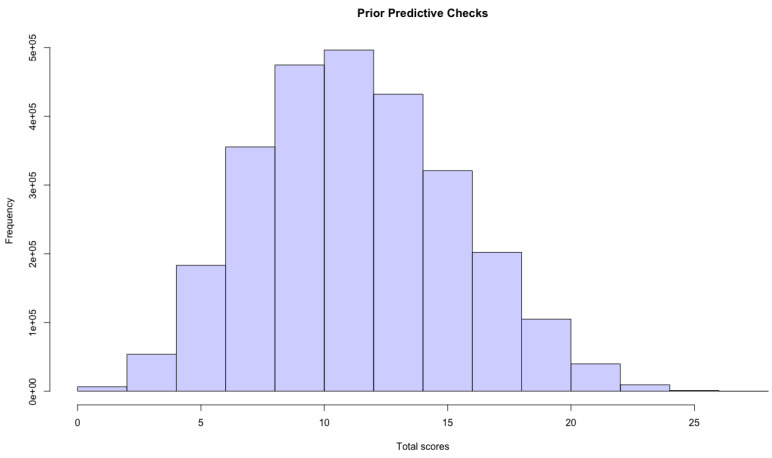
Prior predictive checks.

**Figure A2:**
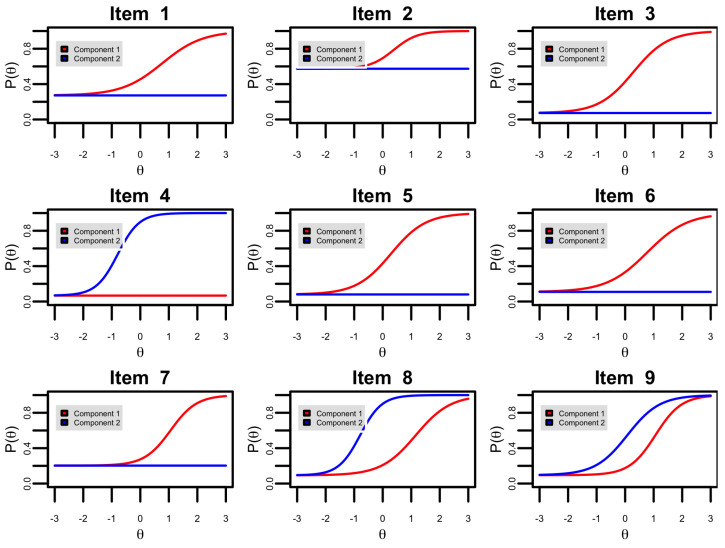

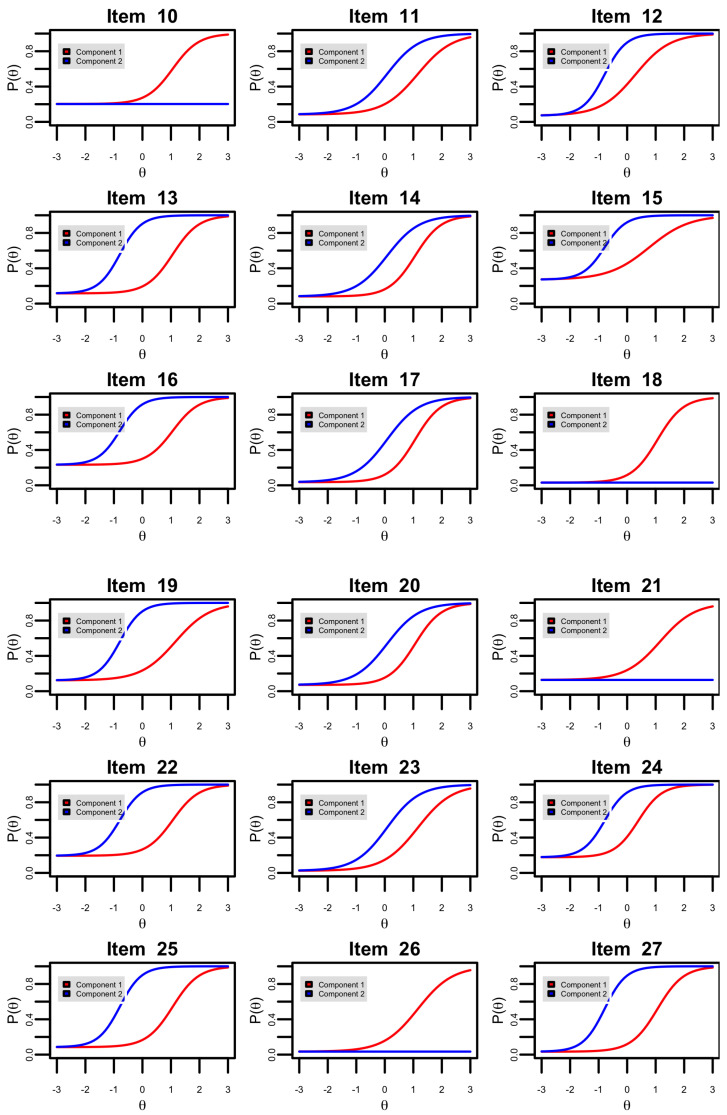
Characteristic curves of the 27 items of the analogical reasoning test.

**Figure A3:**
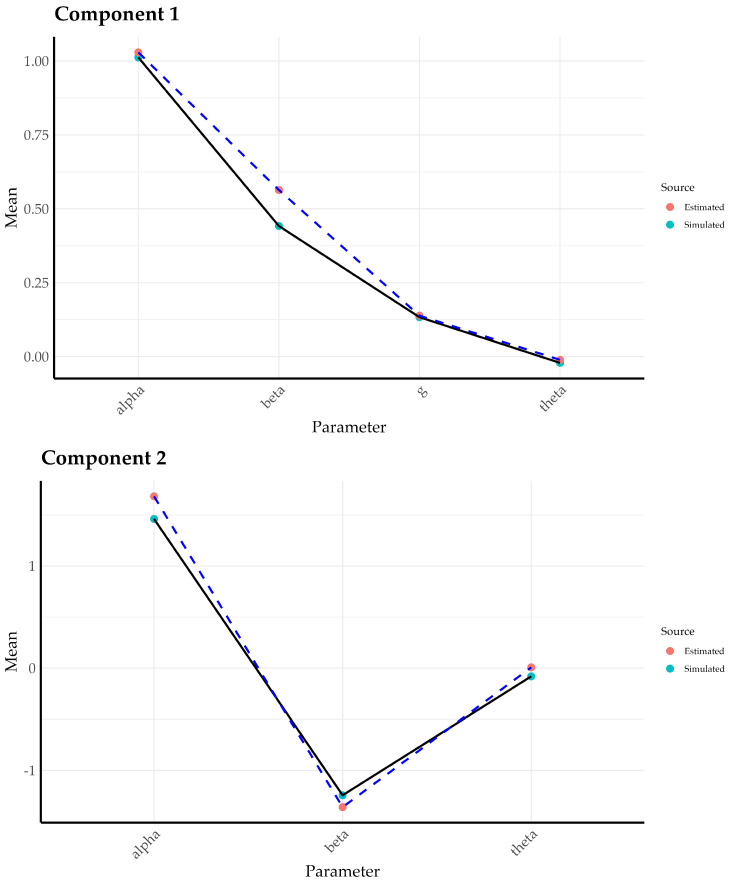
Parameter recovery accuracy in the simulation study. This displays the means of simulated parameters and the parameters recovered by the GMLTM-D across each simulated component. It also illustrates the effectiveness of the GMLTM-D in accurately recovering simulated parameters.

## Data Availability

The data and code for this research are available at https://bit.ly/3xrP3Rq (accessed on 17 July 2024).

## Note

1 In the original parameterization, Embretson and Yang (2013) included an intercept for each component *m*. We omitted these intercepts because are not required for model identification.

## Abbreviations

The following abbreviations are used in this manuscript:

IRT	Item Response Theory
LLTM	Logistic Latent Trait Model
MLTM-D	Multicomponent Latent Trait Model for Diagnosis
GMLTM-D	Generalized Multicomponent Latent Trait Model for Diagnosis

## Appendix C. Parameter Recovery Simulation Study

In this study, the parameter recovery was tested through a simulation study. The data were generated under the GMLTM-D model in the R environment, using the GMLTM package for model estimation. The simulation was carried out by specifying the $C_{jm}$ matrices based on the specification of the *Q* matrix.

In the current simulation, it was assumed that the rules contained in the items were distributed across two components. For all repetitions, the component abilities for 400 examinees were generated from a standard normal distribution θ∼N(0,1). Both the item difficulty and discriminations were simulated considering the model’s definition, so the elements of the η matrix were generated from a standard normal distribution N(0,1). This means that β is a linear combination of elements of η, so β will also follow a normal distribution due to the properties of linear combinations of normal variables. The values of α are generated from a uniform distribution in the interval [1,2.5]. Finally, the guessing parameter (*g*) follows a beta distribution with parameters g∼Beta(3,20).

The generated test consisted of 27 items with the following component structure $C_{jm}$:

$$C_{27\times 2}=\left(\begin{array}{cc} 0 & 1\\ 0 & 1\\ 1 & 0\\ 0 & 1\\ 1 & 0\\ 0 & 1\\ 1 & 1\\ 0 & 1\\ 1 & 1\\ 1 & 1\\ 1 & 1\\ 0 & 1\\ 1 & 1\\ 1 & 1\\ 1 & 1\\ 0 & 1\\ 1 & 1\\ 1 & 1\\ 1 & 0\\ 0 & 1\\ 1 & 1\\ 0 & 1\\ 1 & 1\\ 0 & 1\\ 0 & 1\\ 1 & 1\\ 1 & 1 \end{array}\right)$$

The objective of this simulation was to evaluate the ability of the GMLTM-D to accurately recover the underlying parameters in conditions similar to those of the applied study presented, considering the structure of rules that compose the items, which are hierarchically organized into components. The simulation results provided valuable information about the model’s effectiveness and robustness in parameter recovery in controlled scenarios.

For model estimation using data simulated with GMLTM-D, the Hamiltonian Monte Carlo (HMC) sampling method was employed. The process involved two chains, each running for 500 iterations, utilizing the prior distributions described in the study applied to real data.

Figure A3 illustrates the mean parameter estimates for both components (1 and 2). Remarkably, the simulated and estimated values for each parameter closely align. Although the guessing parameter is estimated globally for the model, it is specifically shown within the first component. The simulation results affirm that under the studied conditions, accurately recovering parameters is feasible using a sample of 400 simulated examinees, mirroring real-world application scenarios.
